# Supplementary material for: Comprehensive benchmark and architectural analysis of deep learning models for nanopore sequencing basecalling
Source: Genome Biol. 2023 Apr 11;24:71. doi: 10.1186/s13059-023-02903-2 (PMC10088207; doi:10.1186/s13059-023-02903-2)
Supplement: Supplementary file 1 — Additional file 1. Figs. S1-S28, Tables S1-S6. [file 13059_2023_2903_MOESM1_ESM.pdf]

List of Supplementary Figures

1 Summary of dataset sizes . . . 2
2 Error profiles of existing basecallers . . . 3
3 Clustering of error profiles . . . 4
4 Benchmark of architecture components . . . 5
5 Comparison of CTC and CRF decoders . . . 6
6 Comparison of simple and complex convolutions . . . 6
7 Comparison of RNN and Transformer encoders . . . 7
8 Comparison of LSTM detph . . . 7
9 Task comparison of cross-species and global benchmarked models . . . 8
10 Dataset difficulty analysis . . . 9
11 Task comparison of human, cross-species and global benchmarked models on human data . . . 10
12 Clustering of benchmark dataset species . . . 11
13 Convolutional architecture of Bonito . . . 12
14 Convolutional architecture of CATCaller . . . 12
15 Convolutional architecture of CausalCall . . . 12
16 Convolutional architecture of Halcyon . . . 13
17 Convolutional architecture of Mincall . . . 14
18 Convolutional architecture of SACall . . . 15
19 Convolutional architecture of URNano . . . 16
20 Encoder architecture of Bonito . . . 17
21 Encoder architecture of BonitoFwd . . . 17
22 Encoder architecture of SACall . . . 18
23 Encoder architecture of CATCaller . . . 18
24 Encoder architecture of URNano . . . 19
25 Encoder architecture of LSTM1 . . . 19
26 Encoder architecture of LSTM3 . . . 19
27 Encoder architecture of LSTM5 . . . 20
28 Performance comparison of recreation and original models . . . 21

List of Supplementary Tables

1 Summary of the collected datasets for benchmarking . . . 22
2 Original models benchmark summary . . . 23
3 Datasets used for basecalling benchmarking . . . 23
4 Species split in the cross-species task . . . 24
5 Recreated models vs original models benchmark summary . . . 25
6 Packages and versions used for model training and evaluation . . . 25

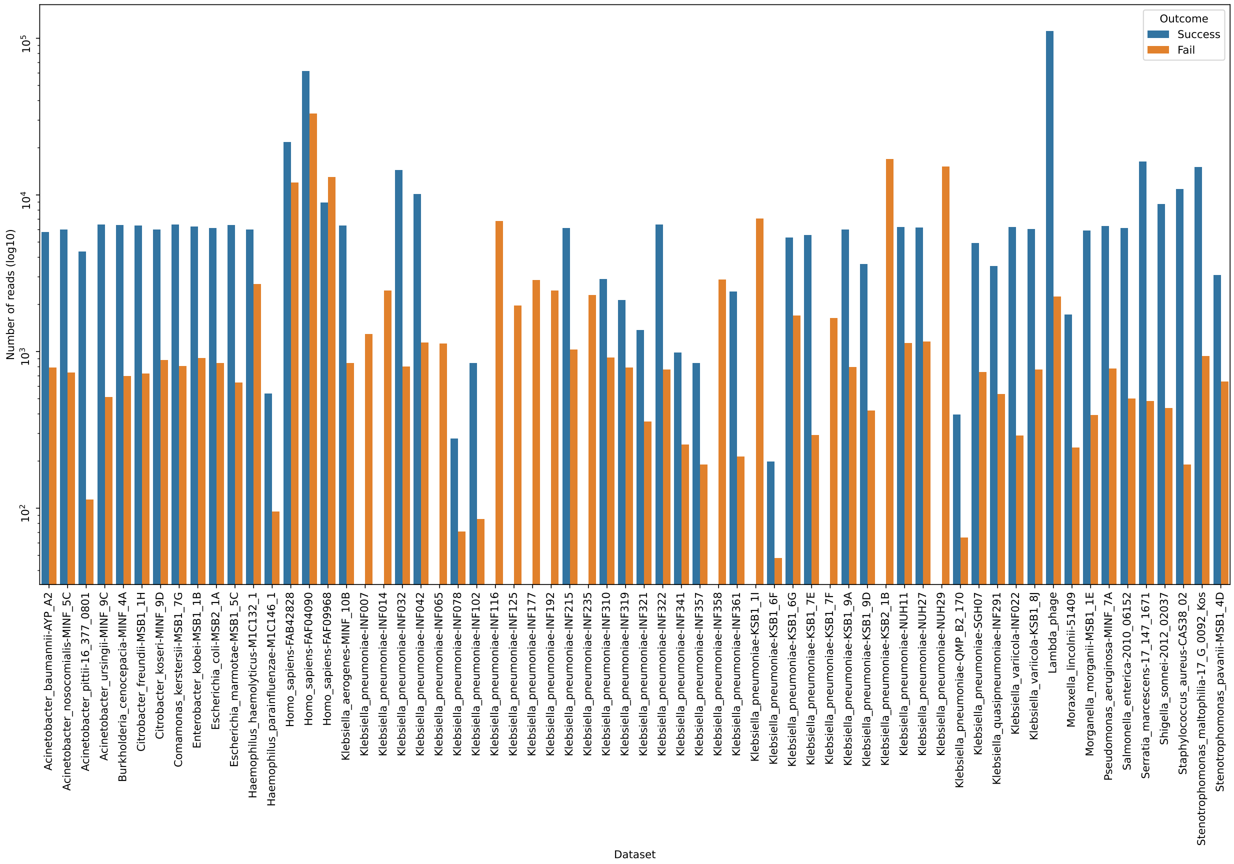

Fig S1: Summary of dataset sizes. Number of reads per dataset that were successfully resquiggled (blue) or that failed (orange).

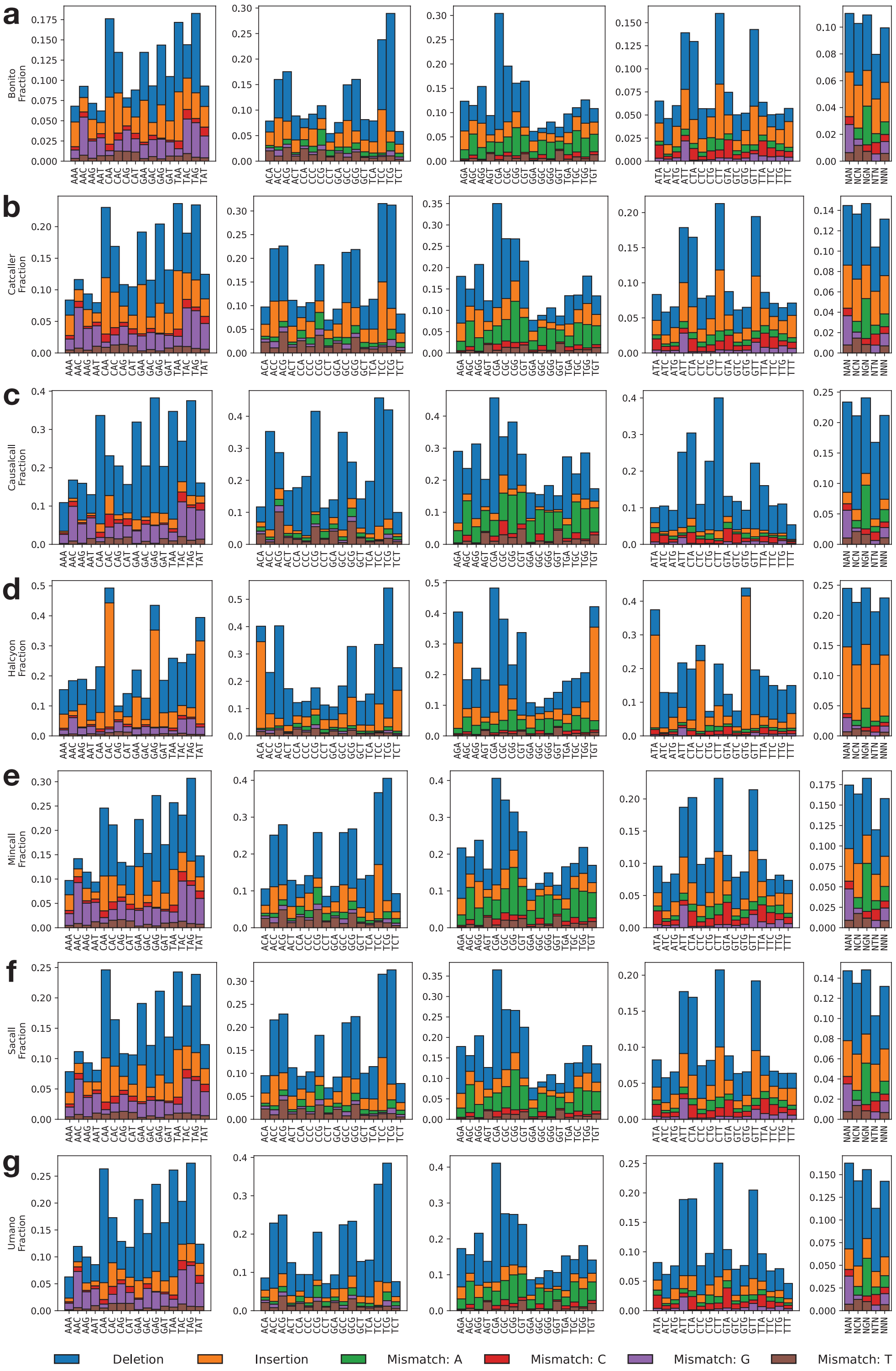

Fig S2: Error profiles of existing basecallers. Error profiles for each benchmarked model. **(a)** Bonito, **(b)** CATCaller, **(c)** Causalcall, **(d)** Halcyon, **(e)** Mincall, **(f)** SACall and **(g)** URNano. Right most panels show error types aggregated by base regardless of context (NAN, NCN, NGN, NTN) or error types regardless of base (NNN).

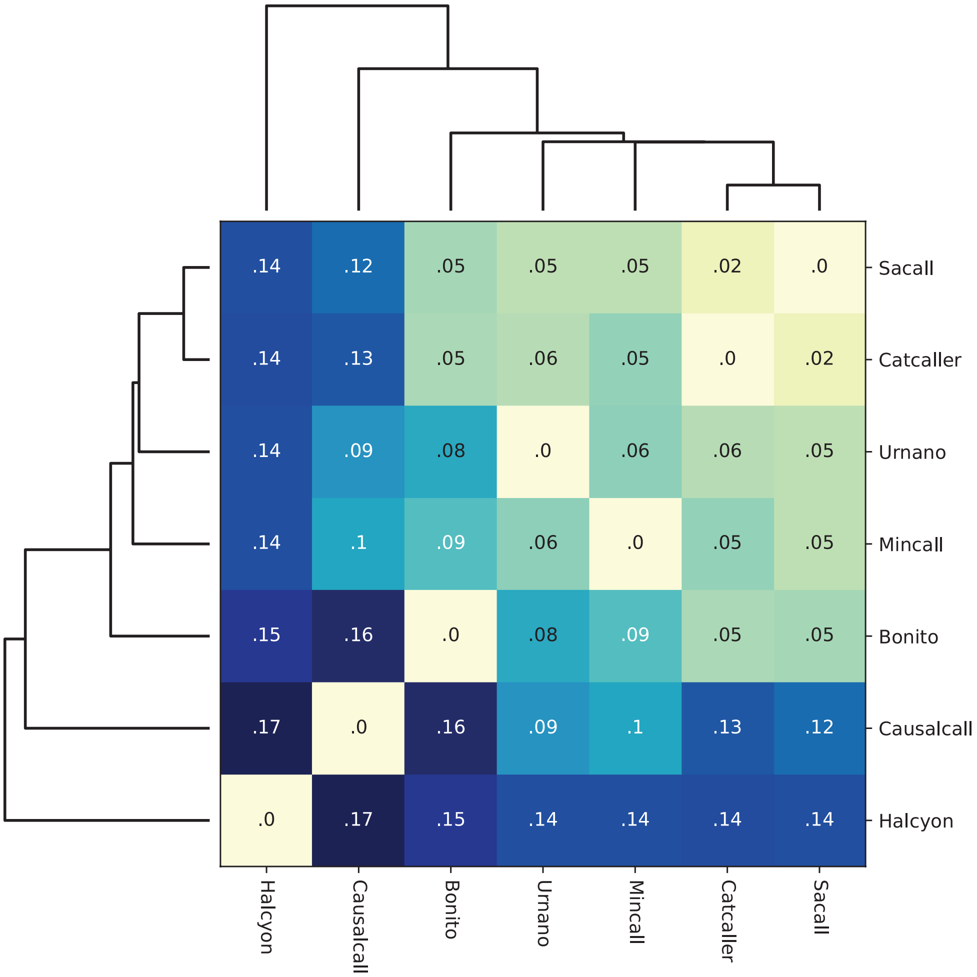

Fig S3: Clustering of error profiles. Hiearchical clustering of the error profiles from the benchmarked basecallers. Values indicate the Jensen-Shannon divergence between profiles.

Convolution	Encoder	Decoder	Evaluated	Failed alignment	Short alignment	No prediction	Match	Mismatch	Insertion	Deletion	Adenine	Cytosine	Guanine	Thymine	AUC	PhredQ overlap
URN	LS5	CRF	93.9	3.68	2.30	0.15	90.1	2.90	2.96	4.08	13.0	15.5	18.9	16.1	91.3	32.8
CAU	LS5	CRF	93.9	3.69	2.25	0.15	90.0	2.91	2.98	4.06	12.8	15.8	19.0	16.1	91.3	33.9
URN	BFW	CRF	93.6	3.94	2.25	0.20	90.0	2.94	2.94	4.14	13.0	16.1	19.3	16.1	91.3	30.3
URN	LS3	CRF	93.8	3.74	2.30	0.15	89.9	2.96	3.04	4.13	13.4	16.0	19.1	16.7	91.2	34.7
SAC	LS5	CRF	93.9	3.52	2.42	0.14	90.0	2.86	2.56	4.55	14.1	15.8	20.0	18.8	91.4	37.2
BON	LS5	CRF	93.7	3.88	2.28	0.16	89.8	2.98	3.06	4.13	13.5	16.2	19.8	16.4	91.1	34.8
BON	BFW	CRF	93.4	4.19	2.24	0.21	89.8	3.00	3.04	4.18	13.1	16.4	19.5	16.8	91.1	32.9
CAT	LS5	CRF	93.7	3.66	2.46	0.18	90.0	2.84	2.55	4.57	13.8	16.1	19.6	18.7	91.4	39.2
BON	BRE	CRF	93.6	3.81	2.37	0.23	89.8	2.98	3.07	4.17	13.1	16.2	19.7	16.6	91.1	36.6
CAT	BFW	CRF	93.5	3.99	2.39	0.16	90.0	2.88	2.59	4.55	13.9	16.1	19.8	17.5	91.3	36.3
URN	BRE	CRF	93.7	3.63	2.39	0.24	89.5	3.11	3.06	4.35	13.4	16.4	20.2	16.9	90.8	35.5
CAU	BFW	CRF	93.5	3.29	2.44	0.74	89.5	3.09	3.08	4.28	13.7	16.5	19.6	16.7	90.9	30.9
SAC	BRE	CRF	93.6	3.74	2.46	0.18	89.9	2.87	2.54	4.69	14.0	16.5	20.8	18.9	91.3	40.6
CAU	BRE	CRF	93.6	3.45	2.45	0.46	89.5	3.07	2.99	4.42	13.9	16.5	20.7	17.4	90.8	36.8
CAU	LS3	CRF	93.5	3.74	2.46	0.30	89.4	3.11	3.04	4.42	13.4	16.8	20.5	17.8	90.7	34.0
SAC	BFW	CRF	93.5	3.56	2.50	0.44	89.6	2.98	2.55	4.83	15.0	16.8	21.0	19.5	91.1	34.2
URN	LS1	CRF	93.4	4.11	2.34	0.18	89.2	3.20	3.24	4.38	13.8	17.3	21.0	17.5	90.6	34.3
BON	LS3	CRF	93.9	3.48	2.35	0.27	89.2	3.20	3.05	4.58	14.1	18.0	21.9	18.2	90.5	35.1
MIN	LS5	CRF	93.5	3.78	2.53	0.16	89.2	3.09	2.61	5.05	14.6	17.6	21.7	19.7	90.8	37.5
MIN	BRE	CRF	93.5	3.79	2.56	0.17	89.0	3.16	2.65	5.16	15.0	17.9	22.6	19.7	90.5	39.6
CAU	LS1	CRF	93.6	3.85	2.42	0.08	88.2	3.51	3.34	4.91	15.2	19.3	23.4	19.7	89.7	33.8
BON	LS1	CRF	93.6	4.00	2.37	0.04	88.0	3.59	3.62	4.76	15.2	19.2	23.7	18.8	89.6	35.6
SAC	LS3	CRF	92.9	3.60	2.76	0.73	89.2	3.07	2.52	5.16	15.7	17.9	22.0	21.0	90.8	35.0
MIN	BFW	CRF	93.4	3.54	2.69	0.40	88.5	3.39	2.76	5.37	16.3	18.7	22.8	20.7	90.1	36.0
CAT	LS3	CRF	92.7	3.78	2.85	0.63	89.3	3.05	2.58	5.11	15.5	17.6	22.2	21.2	90.8	37.6
URN	URN	CRF	93.1	3.86	2.72	0.27	88.4	3.46	3.14	5.02	15.5	19.0	23.3	18.9	89.9	36.9
CAT	BRE	CRF	92.7	4.42	2.74	0.12	88.8	3.07	2.46	5.69	16.9	18.3	24.1	20.9	90.4	43.3
CAU	CAT	CTC	91.3	6.08	2.44	0.18	85.6	4.29	4.99	5.12	13.3	17.6	19.8	15.6	87.8	11.9
URN	CAT	CRF	92.7	4.18	2.69	0.44	88.4	3.46	3.16	5.01	15.8	19.8	23.3	19.4	89.8	35.2
MIN	URN	CRF	93.2	3.88	2.82	0.10	88.1	3.43	2.95	5.52	16.5	20.6	26.2	20.9	89.9	43.0
SAC	LS3	CTC	92.0	4.66	2.45	0.94	86.5	3.92	3.73	5.79	14.6	19.2	21.2	18.1	89.0	8.20
BON	SAC	CTC	91.0	6.52	2.32	0.13	84.3	4.69	5.57	5.38	13.6	18.4	21.8	15.3	86.5	9.14
CAT	LS1	CRF	93.4	3.91	2.67	0.07	87.8	3.54	2.90	5.79	18.0	21.4	28.5	23.9	89.5	34.3
MIN	CAT	CTC	91.8	5.85	2.27	0.08	86.0	4.17	3.75	6.07	14.7	21.2	24.4	17.5	88.4	8.13
SAC	BRE	CTC	92.2	4.19	2.43	1.18	87.0	3.76	3.77	5.45	14.8	19.3	21.8	19.8	89.5	11.2
MIN	LS1	CRF	92.9	3.94	2.84	0.32	88.3	3.40	2.84	5.49	16.5	20.2	26.0	22.3	89.9	37.1
BON	LS5	CTC	90.9	6.00	2.65	0.41	85.0	4.50	5.40	5.06	13.0	17.1	21.2	15.8	87.4	9.06
SAC	LS1	CRF	93.2	4.08	2.63	0.05	87.8	3.56	2.99	5.68	17.7	21.9	27.9	23.3	89.5	35.9
SAC	LS5	CTC	91.5	5.38	2.63	0.48	86.4	3.99	3.97	5.67	14.2	20.0	21.4	18.3	88.9	8.35
BON	BFW	CTC	90.5	6.64	2.62	0.24	84.3	4.85	5.58	5.24	13.4	18.0	21.3	16.4	86.7	7.96
BON	CAT	CTC	90.0	7.48	2.32	0.18	83.5	5.01	5.67	5.84	13.7	17.8	21.5	14.7	85.5	7.96
CAU	URN	CRF	92.6	4.64	2.52	0.24	87.5	3.73	3.53	5.18	15.5	20.2	26.0	19.9	89.3	41.4
BON	CAT	CRF	93.3	4.04	2.59	0.05	87.5	3.79	3.68	5.04	16.2	21.2	28.1	21.5	88.4	46.2
CAT	LS3	CTC	91.4	5.58	2.38	0.60	85.3	4.40	4.39	5.90	14.5	21.2	21.9	17.7	87.8	7.12
CAU	SAC	CTC	90.7	6.73	2.37	0.22	84.2	4.96	5.09	5.78	14.0	20.2	22.7	15.8	86.3	8.10
CAU	CAT	CRF	92.8	4.56	2.48	0.18	87.6	3.73	3.33	5.28	17.4	22.0	27.1	20.3	88.6	40.9
BON	LS3	CTC	89.4	7.42	2.68	0.52	83.3	5.25	6.10	5.37	13.2	19.0	20.6	15.4	85.6	7.47
SAC	CAT	CTC	92.1	5.47	2.32	0.15	86.0	4.07	3.77	6.16	16.0	25.2	29.4	20.2	88.4	8.00
CAT	LS5	CTC	91.2	5.67	2.69	0.45	85.9	4.16	3.87	6.02	14.6	20.7	23.3	18.5	88.5	8.05
CAT	CAT	CRF	92.7	4.39	2.77	0.14	87.9	3.49	3.04	5.58	17.1	23.6	30.1	27.0	89.1	49.0
MIN	BFW	CTC	91.6	4.72	2.50	1.20	85.3	4.50	3.75	6.40	15.0	20.2	23.6	19.4	87.8	5.57
MIN	CAT	CRF	92.7	4.30	2.87	0.08	87.6	3.55	2.95	5.89	18.7	23.3	30.0	23.3	89.0	44.9
URN	LS3	CTC	91.9	4.81	2.92	0.33	87.8	3.04	1.75	7.43	26.3	24.8	30.3	30.9	90.3	10.4
MIN	LS3	CRF	92.4	3.76	2.90	0.90	87.5	3.68	2.83	5.95	18.1	21.2	26.8	22.8	89.3	36.6
MIN	LS5	CTC	91.1	5.63	2.67	0.60	85.1	4.54	3.91	6.42	14.6	21.1	22.9	17.3	87.6	7.99
URN	CAT	CTC	90.5	6.36	2.84	0.26	84.8	4.67	4.78	5.70	14.2	19.1	21.6	16.9	87.2	10.8
BON	BRE	CTC	90.1	6.54	2.86	0.54	84.2	4.88	5.54	5.39	13.5	18.1	21.5	16.6	86.6	12.8
MIN	BRE	CTC	91.6	4.74	2.59	1.03	85.4	4.47	3.91	6.18	14.5	20.7	23.3	19.4	88.0	10.3
CAU	LS5	CTC	91.7	2.59	2.96	2.71	87.2	3.17	1.72	7.93	26.9	27.4	30.2	31.6	89.8	9.64
CAT	SAC	CTC	92.1	4.95	2.38	0.55	86.3	3.93	3.35	6.38	18.7	25.6	28.9	22.8	88.8	8.15
CAU	LS3	CTC	91.7	3.65	2.91	1.71	87.3	3.13	1.68	7.87	25.6	29.4	32.2	31.5	89.9	9.62
SAC	SAC	CTC	91.9	3.44	2.45	2.20	86.1	3.82	3.01	7.07	22.4	26.6	31.3	25.0	88.6	9.23
URN	LS5	CTC	91.7	2.91	3.01	2.40	86.9	3.35	1.88	7.83	28.0	26.8	32.3	31.6	89.5	9.96
MIN	SAC	CTC	90.9	6.61	2.16	0.36	83.4	5.20	4.78	6.63	16.1	22.2	27.9	17.6	85.7	5.67
SAC	SAC	CRF	92.8	3.61	2.54	1.08	87.3	3.73	3.43	5.50	20.1	24.7	30.7	29.9	88.6	47.3
CAT	CAT	CTC	92.0	5.32	2.38	0.32	85.8	4.09	3.65	6.41	16.3	28.0	30.6	20.2	88.3	10.1
BON	URN	CTC	84.4	12.3	3.18	0.14	80.8	6.29	7.09	5.79	13.2	19.0	22.2	15.4	83.1	8.85
SAC	URN	CRF	92.3	4.52	2.89	0.26	86.9	3.83	3.28	5.96	17.6	22.5	29.0	24.2	88.9	45.8
CAU	BRE	CTC	91.4	2.91	3.03	2.70	86.6	3.49	1.98	7.89	29.3	28.1	30.8	31.6	89.3	11.5
CAT	SAC	CRF	92.4	4.11	2.75	0.70	87.5	3.63	3.28	5.59	20.9	24.8	30.4	29.7	88.7	48.0
BON	LS1	CTC	81.8	15.0	3.14	0.06	79.5	6.78	6.66	7.08	12.9	20.6	22.9	14.4	81.6	8.20
BON	URN	CRF	92.2	4.25	2.71	0.86	86.6	4.09	3.76	5.57	16.7	23.4	28.4	21.4	88.3	41.4
MIN	LS1	CTC	87.7	9.14	3.04	0.08	83.2	5.23	4.49	7.06	15.2	23.1	24.2	17.7	85.9	8.08
URN	BFW	CTC	91.3	2.38	3.10	3.25	86.1	3.66	2.03	8.22	26.7	29.4	32.0	33.0	88.7	9.10
SAC	BFW	CTC	89.5	7.12	2.82	0.60	83.9	5.01	4.48	6.61	15.6	22.4	25.2	18.9	86.6	5.83
CAT	LS1	CTC	84.8	12.2	3.00	0.04	81.1	5.97	5.19	7.68	15.4	23.6	24.6	16.6	83.6	5.62
URN	BRE	CTC	91.2	3.26	3.02	2.54	85.9	3.79	2.17	8.07	26.6	28.6	32.2	34.3	88.7	8.32
SAC	LS1	CTC	85.7	11.3	2.96	0.03	81.7	5.70	5.13	7.43	14.9	22.9	25.5	16.1	84.2	9.10
MIN	LS3	CTC	88.0	9.08	2.79	0.18	82.4	5.60	4.72	7.29	15.2	23.8	26.4	17.4	84.8	7.07
URN	LS1	CTC	90.8	4.88	3.17	1.16	86.1	3.63	1.98	8.31	26.4	29.8	32.0	33.5	88.8	7.24
CAT	BFW	CTC	88.8	7.95	2.88	0.39	83.2	5.31	4.76	6.67	15.9	23.3	25.1	19.6	85.9	5.71
CAT	URN	CRF	91.5	4.64	3.28	0.61	86.5	3.84	3.25	6.39	16.6	24.4	30.9	22.5	88.7	46.8
BON	SAC	CRF	91.3	3.08	2.80	2.82	84.1	5.07	5.22	5.60	19.5	21.1	24.9	27.4	86.3	38.9
CAT	URN	CTC	86.9	10.2	2.82	0.10	81.6	5.88	5.48	6.99	16.1	26.1	29.0	17.7	84.1	6.08
CAU	BFW	CTC	89.9	3.04	3.67	3.41	85.2	3.63	1.81	9.32	32.3	30.0	34.4	36.6	88.1	8.31
SAC	URN	CTC	87.3	9.78	2.76	0.11	81.8	5.82	5.06	7.31	16.7	27.8	26.3	19.0	84.3	6.79
MIN	URN	CTC	81.5	15.2	3.23	0.05	79.7	6.75	5.32	8.24	16.0	27.2	31.0	17.2	82.0	4.69
CAU	LS1	CTC	89.0	6.56	3.80	0.64	84.9	3.70	1.74	9.63	30.2	35.4	37.8	32.9	87.8	7.19
URN	URN	CTC	89.8	5.40	3.40	1.42	84.9	4.05	2.17	8.87	29.5	30.7	32.7	33.4	87.7	7.06
CAT	BRE	CTC	87.9	8.14	3.03	0.94	82.8	5.50	4.82	6.91	16.4	23.7	28.1	19.5	85.5	10.1
CAU	URN	CTC	88.2	5.74	3.83	2.20	83.8	4.08	1.88	10.3	35.0	34.7	42.3	35.8	86.6	6.68

Fig S4: Benchmark of architecture components. Performance of all combined components benchmarked on the human task. Models sorted based on their overall performance ranking. Gradient coloring indicates: good performance (green), average performance (yellow), bad performance (red). Top three models per metric are indicated in gold (1st), silver (2nd) and bronze (3rd) colors.

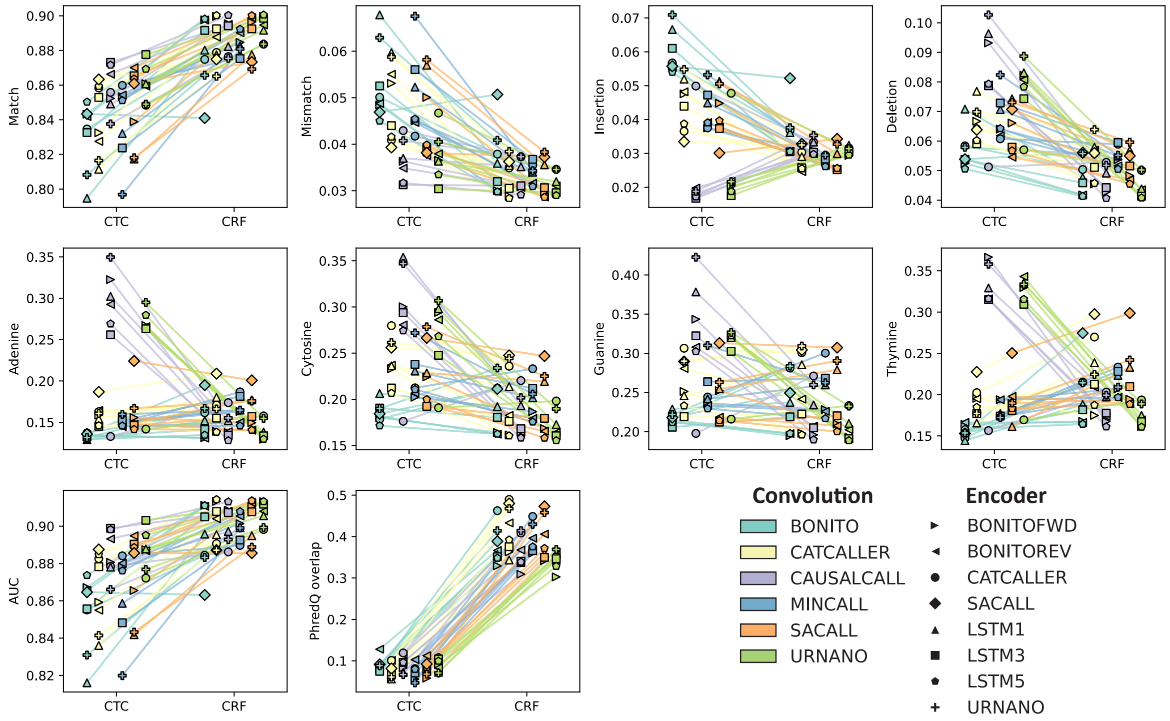

Fig S5: Comparison of CTC and CRF decoders. Pair-wise performance comparison of models that share the same architecture with the exception of the decoder (CTC or CRF).

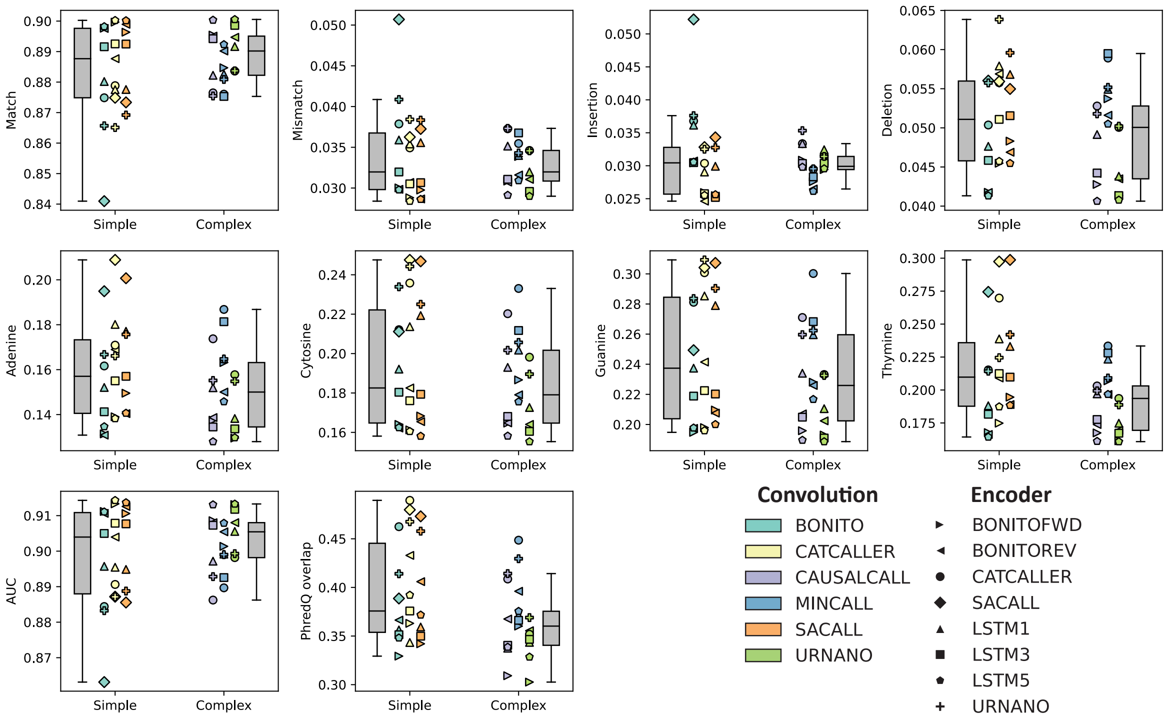

Fig S6: Comparison of simple and complex convolutions. Performance comparison of CRF models that use a simple (*Bonito*, *CATCAller*, *SACall*) vs a complex (*Causalcall*, *Mincall*, *URNano*) convolution.

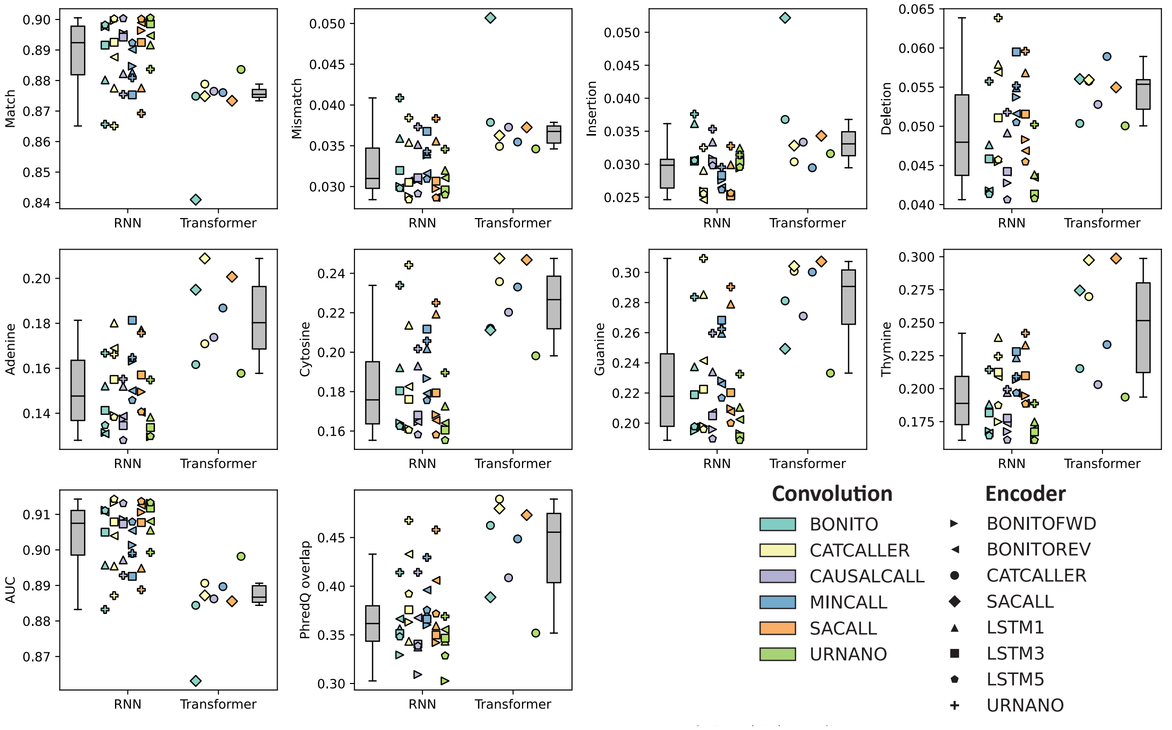

Fig S7: Comparison of RNN and Transformer encoders. Performance comparison of CRF models that use a RNN encoder vs a Transformer encoder

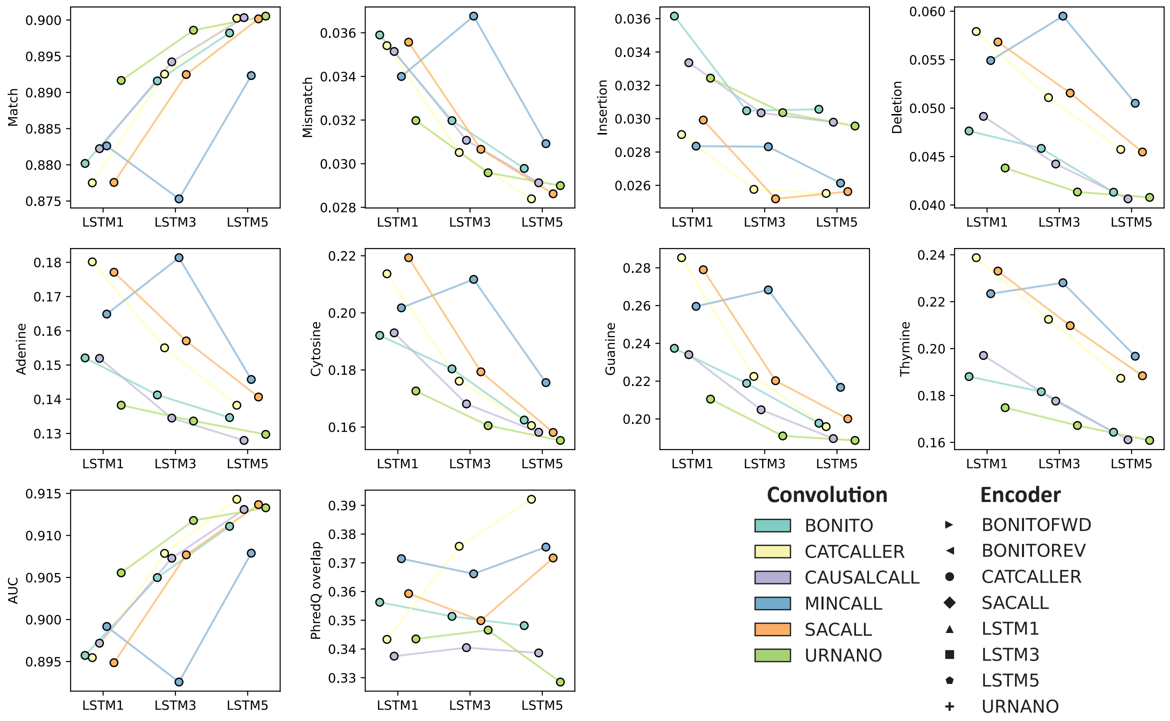

Fig S8: Comparison of LSTM detph. Performance comparison of models with LSTM encoders with different layer depths (1, 3 or 5).

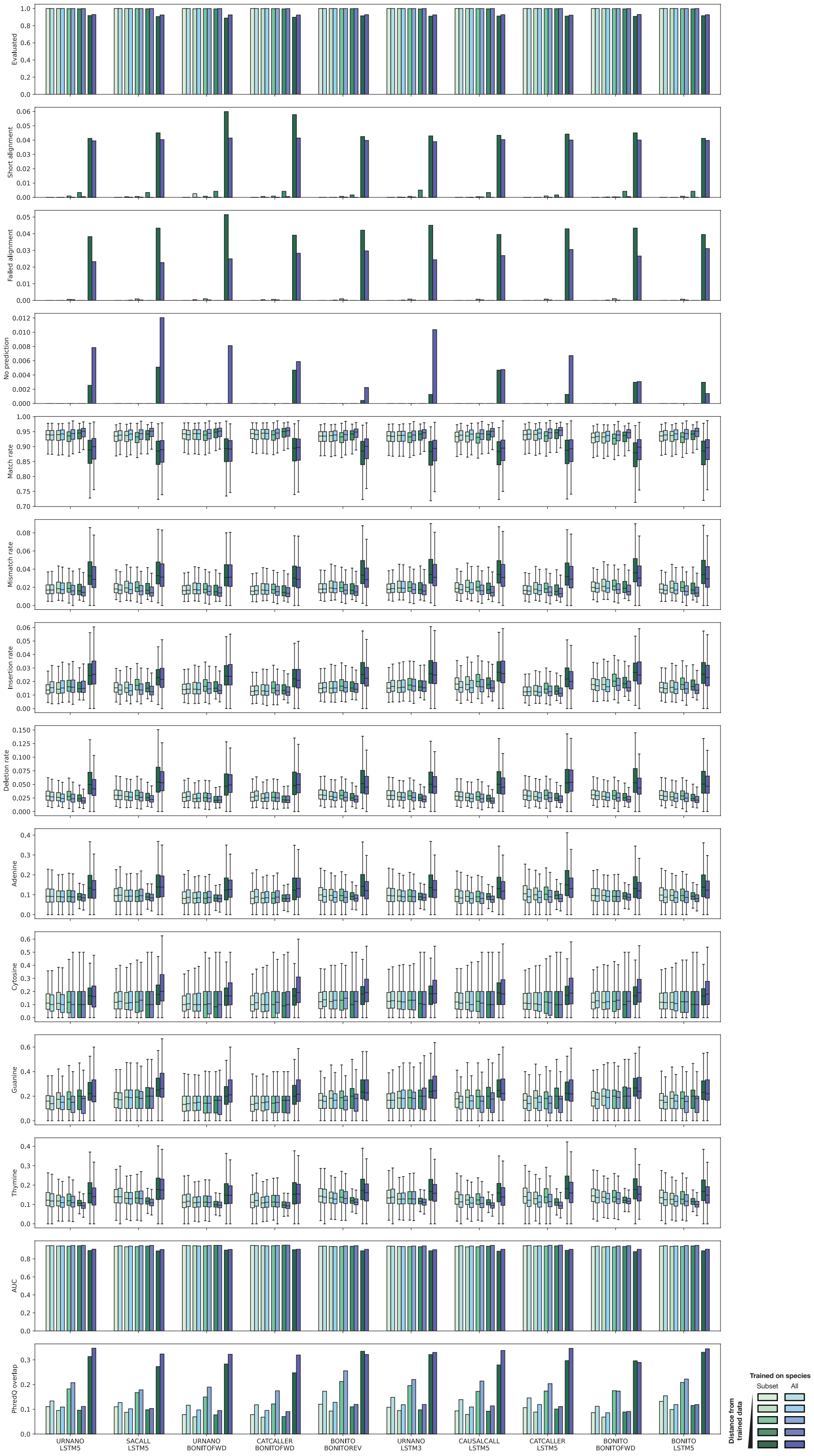

Fig S9: Task comparison of cross-species and global benchmarked models. Comparison of the top 10 model combinations on trained on the cross-species or global datasets and tested on all species binned in the same manner as the cross-species dataset. Darker color indicates species are more different from the train set in the cross-species task.

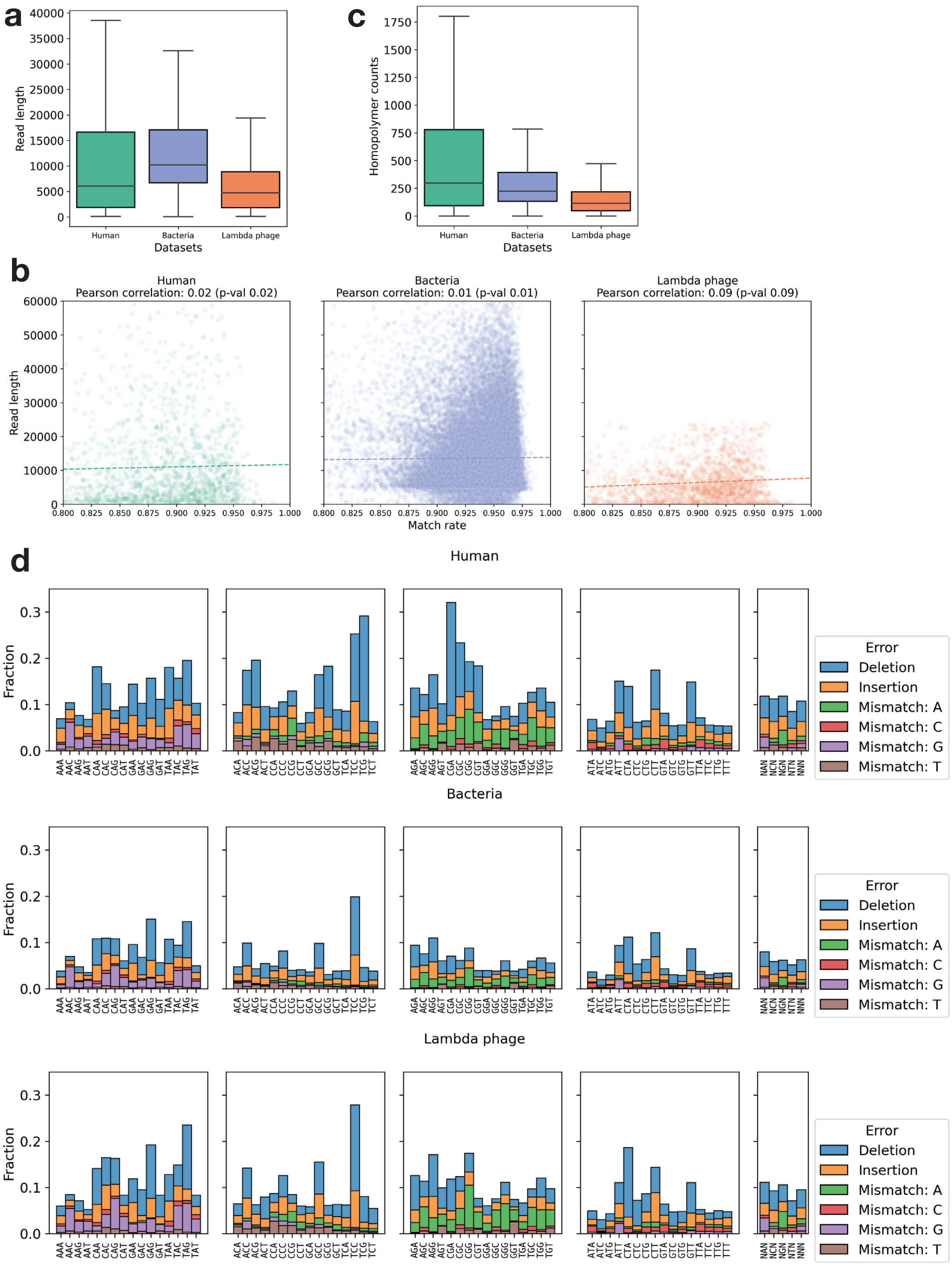

Fig S10: Dataset difficulty analysis. We combined the results of the top 10 models for the global task and analyzed the differences in the 3 datasets (test fold): human, bacteria and lambda phage. (**a**) Read length distribution. **b**) Correlation between read length distribution and match rate per read, title indicates Pearson correlation coefficient and p-value. (**c**) Number of homopolymers per read in each dataset (homopolymers are defined as sequences of the same base of length 5 or longer). (**d**) Error profiles of each dataset in 3-mers.

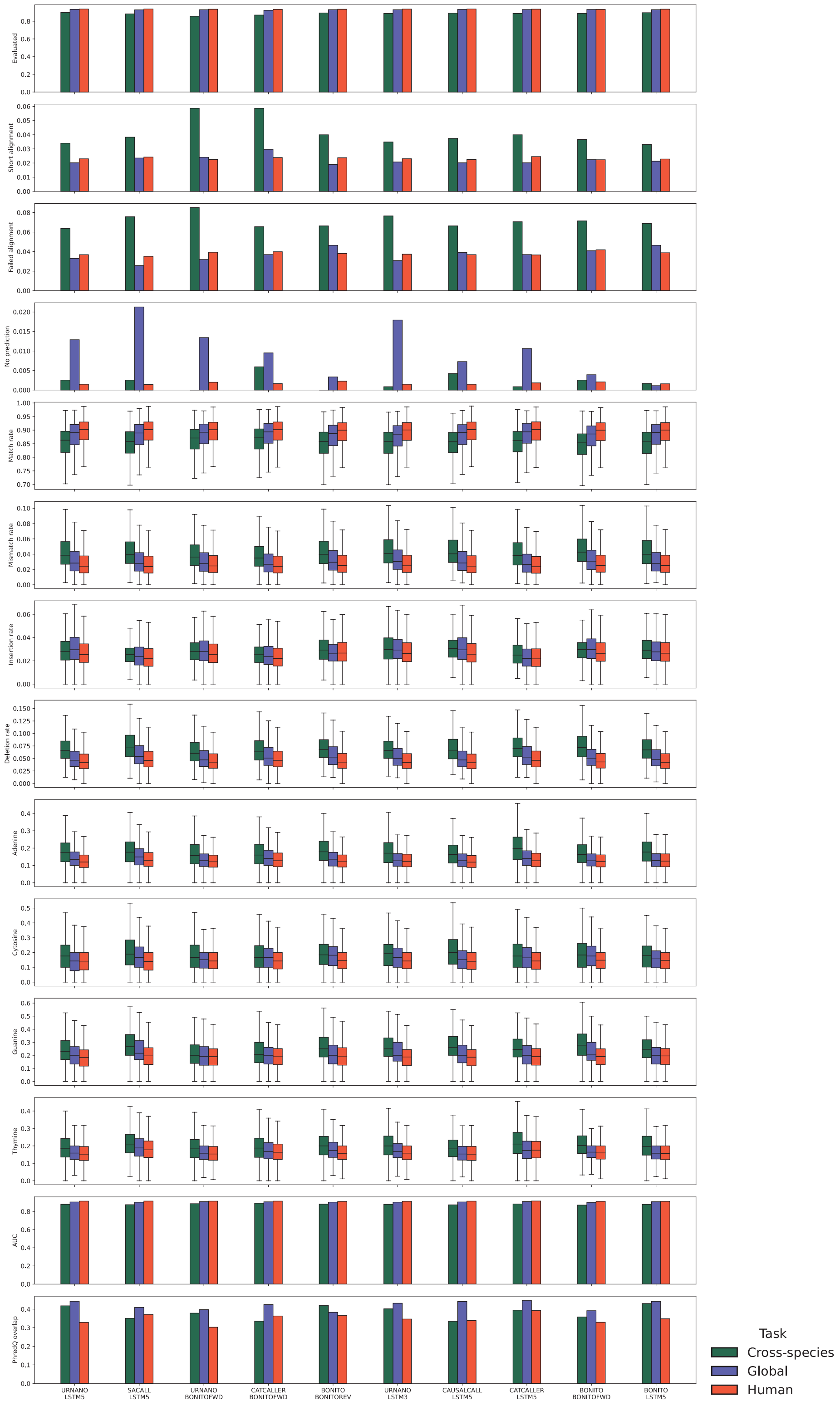

Fig S11: Task comparison of human, cross-species and global benchmarked models on human data. Comparison of the top 10 model combinations on trained on the human, cross-species or global datasets and tested only on human data.

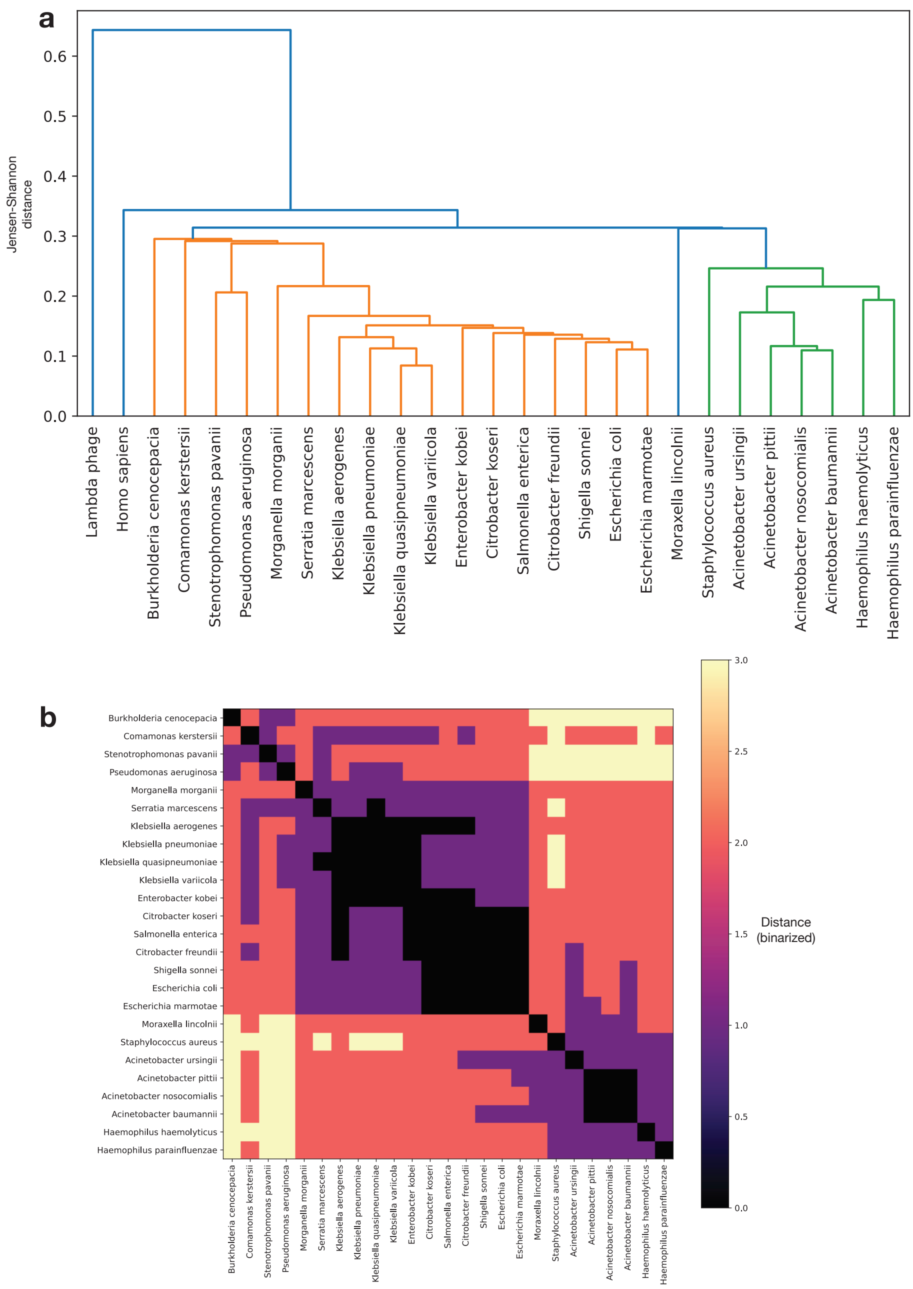

Fig S12: Clustering of benchmark dataset species. (a Single-linkage hierarchical clustering of all the species based on the Jensen-Shannon divergence between the 9-mer relative counts of their genomes. **b** Heatmap with the binned distance between all bacterial species.

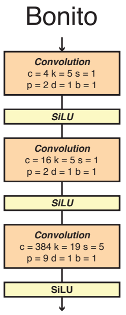

Fig S13: Convolutional architecture of Bonito.

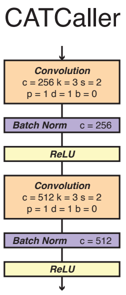

Fig S14: Convolutional architecture of CATCaller.

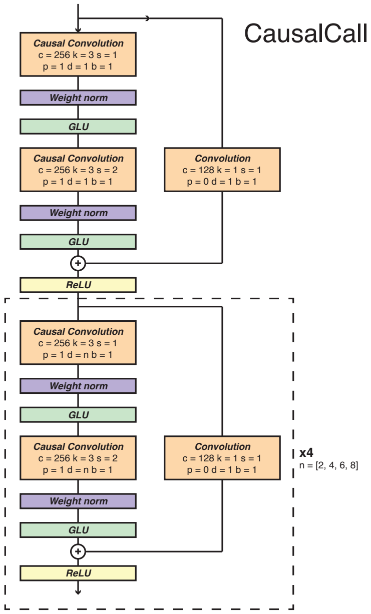

Fig S15: Convolutional architecture of CausalCall.

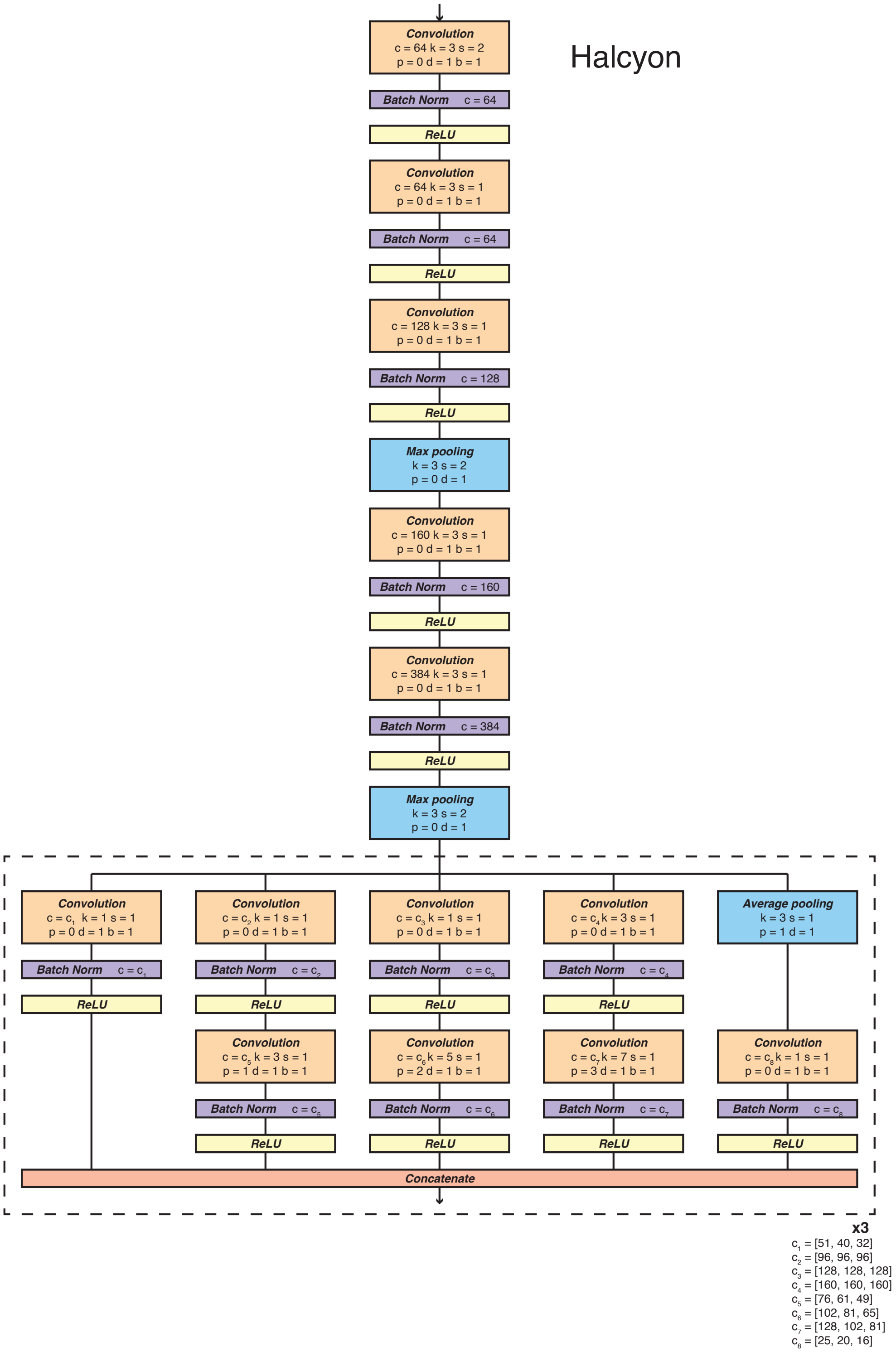

Fig S16: Convolutional architecture of Halcyon.

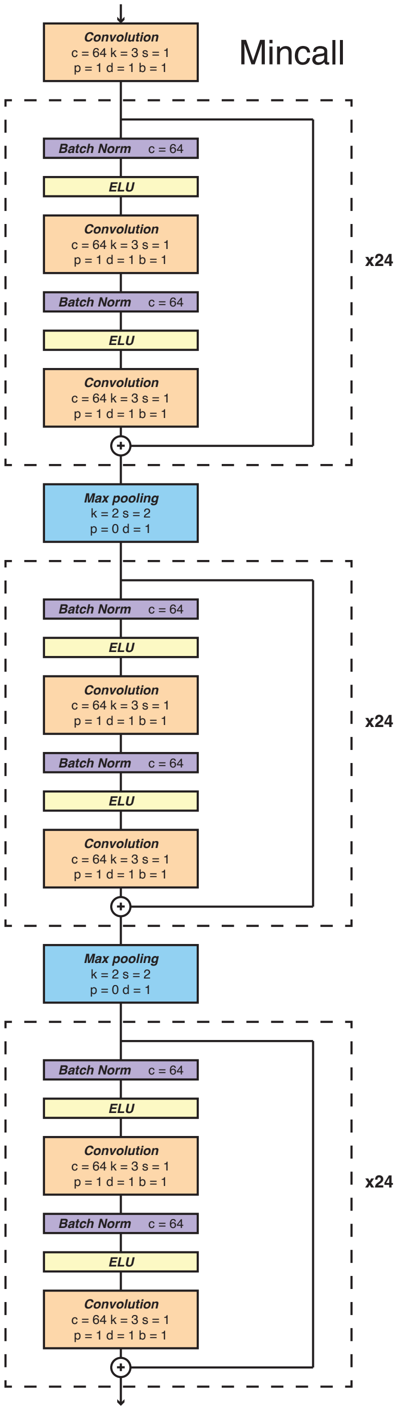

Fig S17: Convolutional architecture of Mincall.

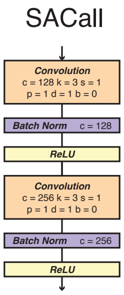

Fig S18: Convolutional architecture of SACall.

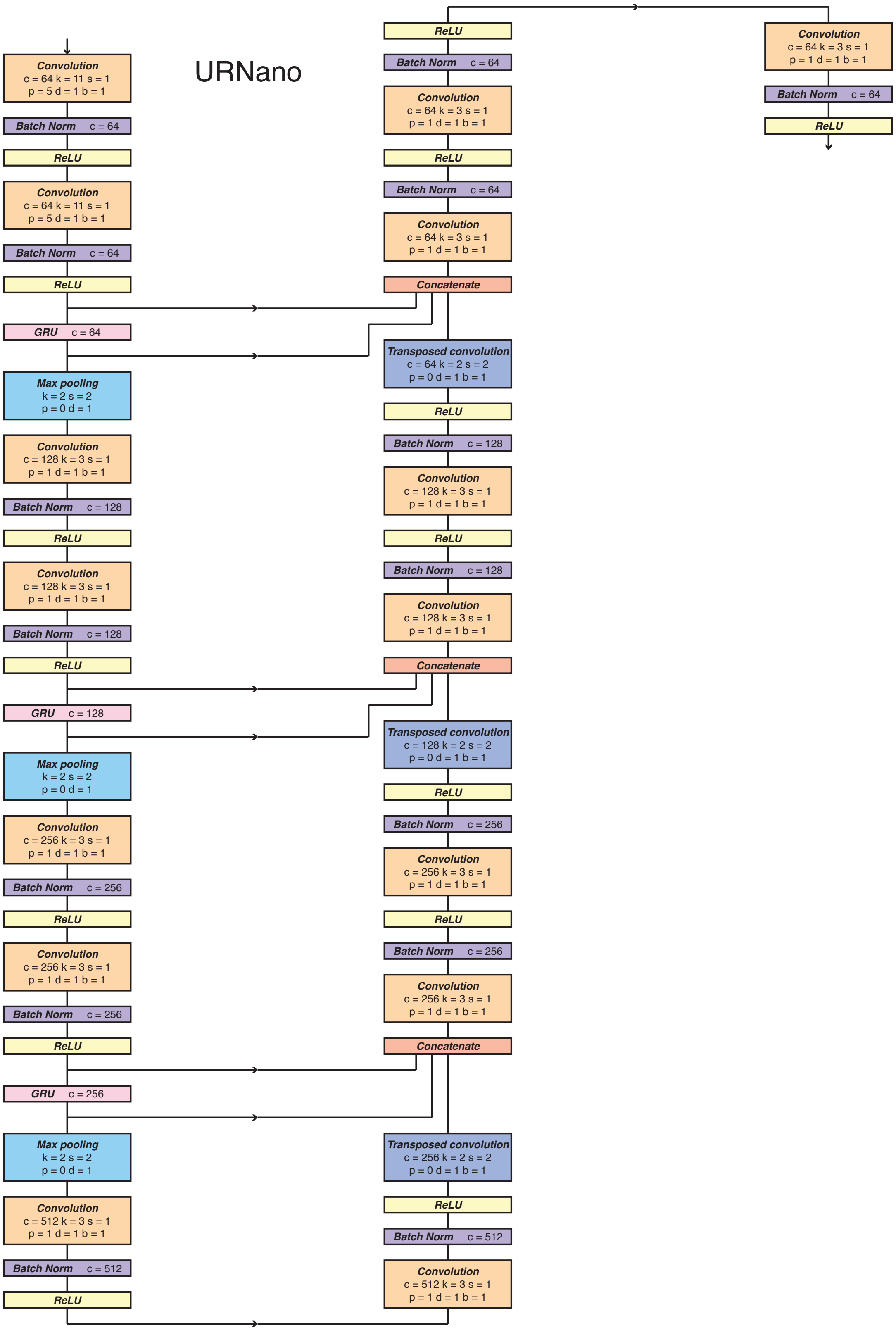

Fig S19: Convolutional architecture of URNano.

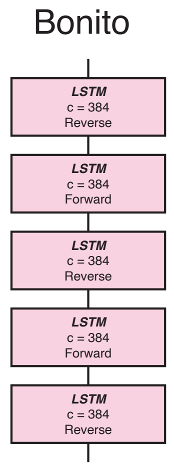

Fig S20: Encoder architecture of Bonito.

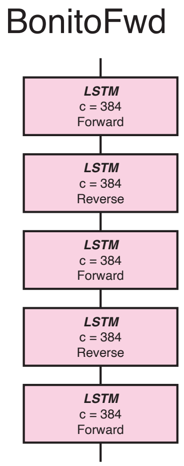

Fig S21: Encoder architecture of BonitoFwd.

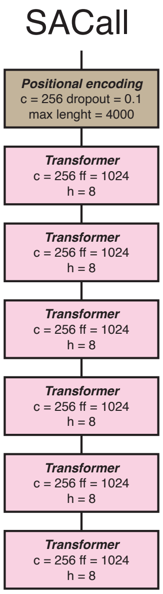

Fig S22: Encoder architecture of SACall.

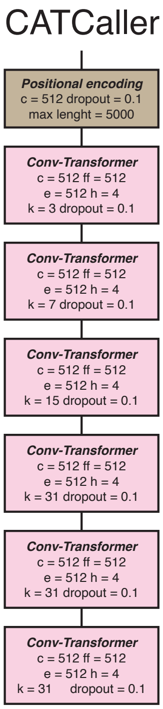

Fig S23: Encoder architecture of CATCaller.

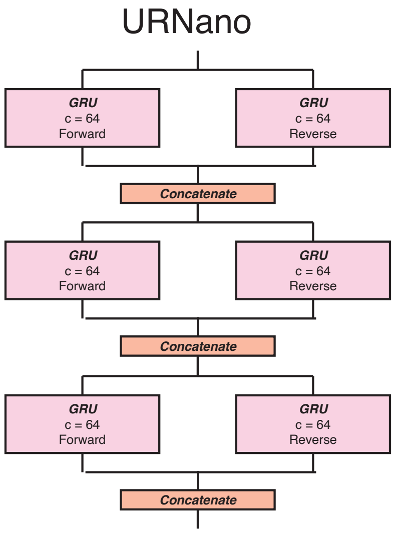

Fig S24: Encoder architecture of URNano.

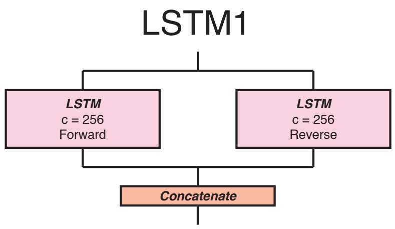

Fig S25: Encoder architecture of LSTM1.

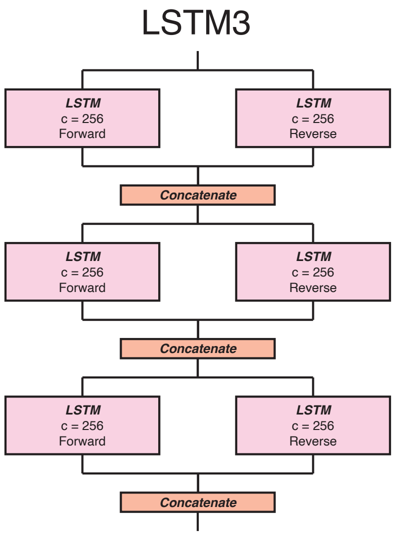

Fig S26: Encoder architecture of LSTM3.

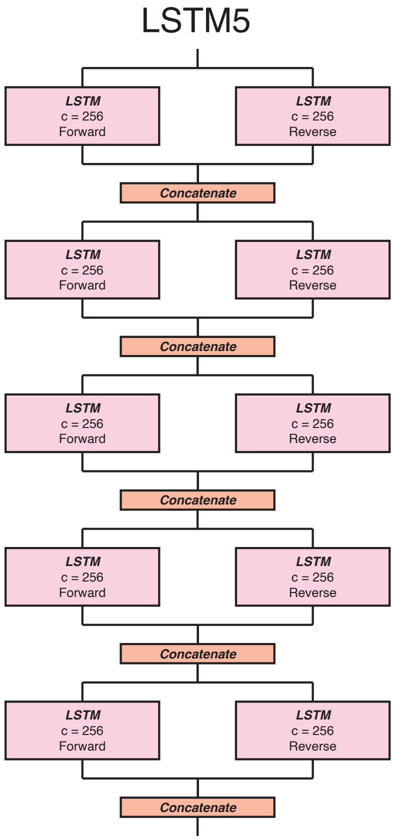

Fig S27: Encoder architecture of LSTM5.

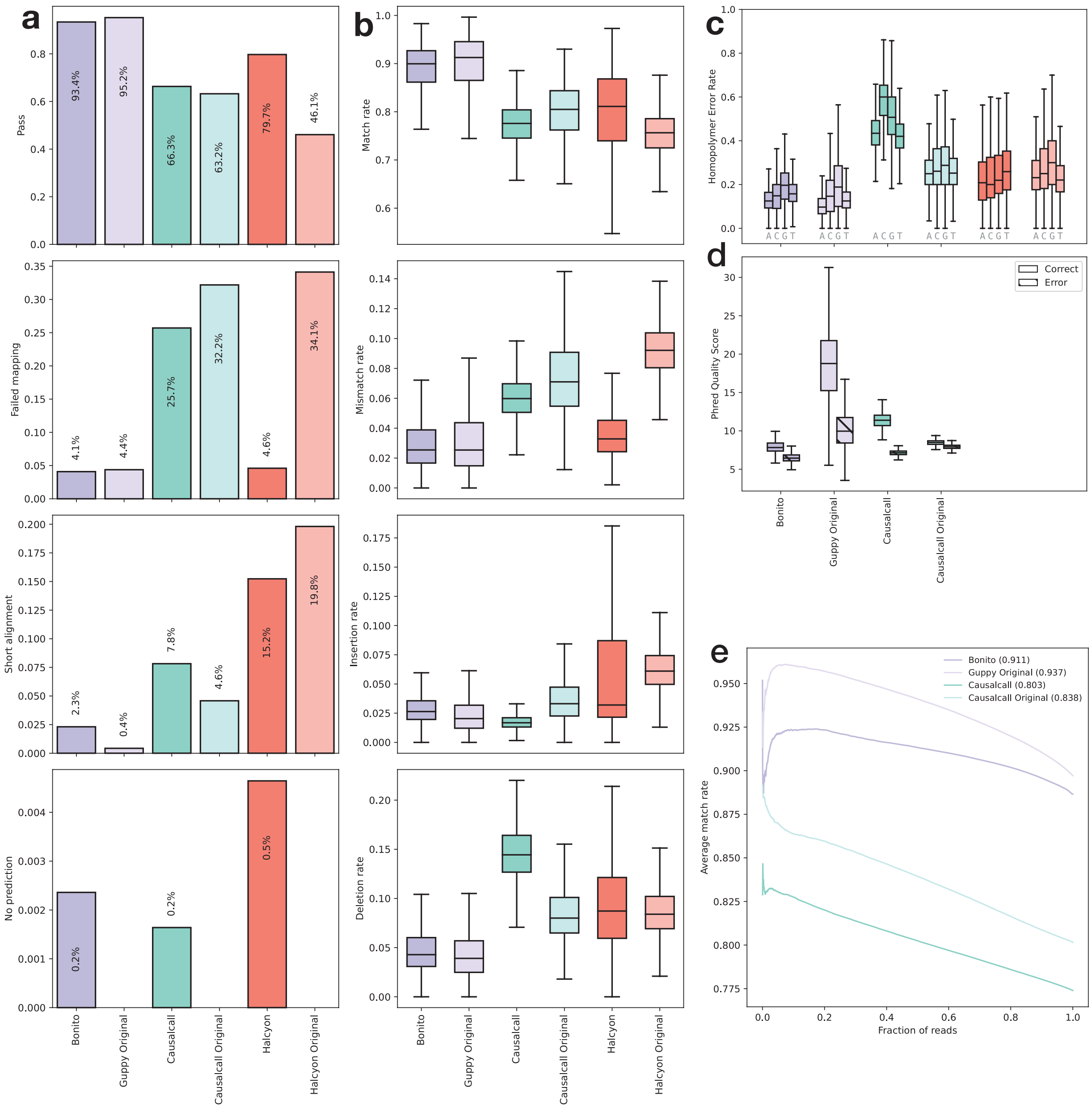

Fig S28: Performance comparison of recreation and original models. Benchmark of our of *Guppy*, *Causalcall*, *Halcyon* recreations and their original counterparts on the human task. **(a)** basecalling reads failure rates: pass, failed mapping, short alignment and no prediction (from top top bottom). **(b)** alignment event rates: match, mismatch, insertion and deletion (from top to bottom). **(c)** homopolymer error rates per base. **(d)** PhredQ scores distributions for correctly (light) and incorrectly (dark) predicted bases. **(e)** AUC of match rate sorted by average read PhredQ score.

Species	Dataset	Total reads	Benchmark reads	Global Train	Global Test	Human Train	Human Test	Cross-species Train	Cross-species Test
Acinetobacter baumannii	AYP-A2	6558	5768	3002	1785	0	0	0	1176
Acinetobacter nosocomialis	MINF-5C	6722	5992	3153	1785	0	0	0	1176
Acinetobacter pittii	16-377-0801	4467	4355	2193	1785	0	0	0	1176
Acinetobacter ursingii	MINF-9C	6976	6466	3069	1785	0	0	0	1176
Burkholderia cenocepacia	MINF-4A	7096	6401	3217	1785	0	0	0	1176
Citrobacter freundii	MSB1-1H	7093	6372	3244	1785	0	0	0	1176
Citrobacter koseri	MINF-9D	6900	6020	3003	1785	0	0	5000	500
Comamonas kerstersii	MSB1-7G	7242	6439	3446	1785	0	0	5000	500
Enterobacter kobei	MSB1-1B	7199	6291	3163	1785	0	0	5000	500
Escherichia coli	MSB2-1A	6985	6140	3157	1785	0	0	0	1176
Escherichia marmotae	MSB1-5C	7064	6431	3167	1785	0	0	0	1176
Haemophilus haemolyticus	M1C132-1	8669	5984	2962	1785	0	0	0	1176
Haemophilus parainfluenzae	M1C146-1	633	539	269	268	0	0	0	539
Homo sapiens	FAB42828	33633	21666	862	398	9935	5836	0	287
	FAF04090	94833	61778	2360	1206	28582	16803	0	792
	FAF09968	21947	8942	349	181	4295	2361	0	97
Klebsiella aerogenes	MINF-10B	7200	6360	3150	1785	0	0	5000	500
Klebsiella pneumoniae	INF007	1287	0	0	0	0	0	0	0
	INF014	2449	0	0	0	0	0	0	0
	INF032	15154	14353	0	0	0	0	834	91
	INF042	11278	10141	0	0	0	0	583	53
	INF065	1120	0	0	0	0	0	0	0
	INF078	347	277	14	4	0	0	23	2
	INF102	928	844	50	22	0	0	55	2
	INF116	6775	0	0	0	0	0	0	0
	INF125	1966	0	0	0	0	0	0	0
	INF177	2853	0	0	0	0	0	0	0
	INF192	2449	0	0	0	0	0	0	0
	INF215	7142	6114	338	194	0	0	379	37
	INF235	2282	0	0	0	0	0	0	0
	INF310	3803	2892	166	105	0	0	175	19
	INF319	2913	2127	118	45	0	0	131	9
	INF321	1729	1374	91	41	0	0	85	7
	INF322	7212	6446	351	189	0	0	350	44
	INF341	1237	984	59	24	0	0	54	9
	INF357	1032	843	46	18	0	0	60	1
	INF358	2876	0	0	0	0	0	0	0
	INF361	2628	2416	140	63	0	0	139	10
	KSB1-1I	7031	0	0	0	0	0	0	0
	KSB1-6F	245	198	13	10	0	0	5	0
	KSB1-6G	7040	5351	333	141	0	0	278	30
	KSB1-7E	5832	5540	297	177	0	0	288	32
	KSB1-7F	1636	0	0	0	0	0	0	0
	KSB1-9A	6787	5991	354	158	0	0	334	29
	KSB1-9D	4043	3624	187	106	0	0	207	19
	KSB2-1B	16847	0	0	0	0	0	0	0
	NUH11	7336	6208	341	174	0	0	353	46
	NUH27	7321	6169	389	149	0	0	367	36
	KNUH29	15178	0	0	0	0	0	0	0
	QMP-B2-170	459	395	28	10	0	0	17	1
	SGH07	5645	4907	256	155	0	0	283	23
Klebsiella quasipneumoniae	INF291	4047	3513	1812	1689	0	0	3013	500
Klebsiella variicola	INF022	6501	6211	1810	877	0	0	2555	262
	KSB1-8J	6806	6039	1761	908	0	0	2445	238
Lambda phage	VER5940	113514	111276	3571	1785	0	0	0	1176
Moraxella lincolnii	51409	1957	1715	731	865	0	0	0	1176
Morganella morganii	MSB1-1E	6307	5915	2866	1785	0	0	5000	500
Pseudomonas aeruginosa	MINF-7A	7082	6307	3106	1785	0	0	0	1176
Salmonella enterica	2010-06152	6638	6138	3142	1785	0	0	5000	500
Serratia marcescens	17-147-1671	16742	16262	3571	1785	0	0	5000	500
Shigella sonnei	2012-02037	9145	8711	3571	1785	0	0	0	1176
Staphylococcus aureus	CAS38-02	11047	10858	3571	1785	0	0	0	1176
Stenotrophomonas maltophilia	17-G-0092-Kos	16010	15075	3571	1785	0	0	0	0
Stenotrophomonas pavanii	MSB1-4D	3706	3067	1535	1426	0	0	0	1176
-	**Total**	**615579**	**460225**	**81955**	**47088**	**42812**	**25000**	**48013**	**24355**

Table S1: Summary of the collected datasets for benchmarking. Collection of datasets used in this benchmark. Benchmark reads indicate the number of reads that could be aligned to their reference sequence. Number of reads used in each task for training and testing.

Model	Basecalled reads (%)				Alignment events (%)				Hompolymer errors (%)				PhredQ scoring	
	Pass	Failed mapping	Short alignment	No prediction	Match	Mismatch	Insertion	Deletion	Adenine	Cytosine	Guanine	Thymine	Overlap	AUC
Bonito	**93.4**	4.1	2.3	0.2	**90.0**	**2.5**	2.6	**4.3**	**12.5**	**14.9**	**19.7**	**15.8**	32.4	**0.910**
CATCaller	92.4	5.2	**2.2**	0.2	86.5	3.6	3.6	5.9	14.2	23.5	27.4	18.2	8.2	0.886
Causalcall	66.3	25.7	7.8	0.2	77.6	6.0	**1.7**	14.4	43.4	60.0	50.7	42.0	**0.7**	0.802
Halcyon	79.5	4.7	15.4	0.4	81.3	3.3	3.2	8.8	20.8	20.0	22.0	25.8	11.7	0.844
Mincall	87.5	9.4	3.1	**0.0004**	83.7	4.9	3.7	7.2	17.2	21.8	28.6	20.0	6.6	0.863
SACall	92.2	**3.7**	2.4	1.7	86.5	3.5	3.0	6.5	20.0	22.6	29.6	19.7	8.7	0.886
URNano	90.4	4.8	3.3	1.6	85.4	3.6	2.0	8.6	28.8	28.6	35.6	32.6	7.2	0.879

Table S2: Original models benchmark summary. Summary of the benchmark on training and testing the latest published basecallers on the human task. Results in bold denote best performance.

Reference	Species	Dataset name	Pore version	Ligation kit	Available
[27]	*Klebsiella pneumoniae*	INF007	R9.4	-	Yes
		INF014	R9.4	-	Yes
		INF065	R9.4	-	Yes
		INF078	R9.4	-	Yes
		INF102	R9.4.1	-	Yes
		INF116	R9.4	-	Yes
		INF125	R9.4	-	Yes
		INF177	R9.4	-	Yes
		INF192	R9.4	-	Yes
		INF215	R9.4	-	Yes
		INF235	R9.4	-	Yes
		INF310	R9.4.1	-	Yes
		INF319	R9.4.1	-	Yes
		INF321	R9.4.1	-	Yes
		INF322	R9.4	-	Yes
		INF341	R9.4.1	-	Yes
		INF357	R9.4.1	-	Yes
		INF358	R9.4	-	Yes
		INF361	R9.4.1	-	Yes
		KSB1-1I	R9.4	-	Yes
		KSB1-6F	R9.4	-	Yes
		KSB1-6G	R9.4	-	Yes
		KSB1-7E	R9.4	-	Yes
		KSB1-7F	R9.4	-	Yes
		KSB1-9A	R9.4	-	Yes
		KSB1-9D	R9.4	-	Yes
		NUH11	R9.4	-	Yes
		NUH27	R9.4	-	Yes
		QMP-B2-170	R9.4	-	Yes
		SGH07	R9.4	-	Yes
	Citrobacter freundii	MSB1-1H	R9.4.1	-	Yes
	Citrobacter koseri	MINF-9D	R9.4.1	-	Yes
	Enterobacter kobei	MSB1-1B	R9.4.1	-	Yes
	Escherichia coli	MSB2-1A	R9.4.1	-	Yes
	Escherichia marmotae	MSB1-5C	R9.4.1	-	Yes
	Klebsiella aerogenes	MINF-10B	R9.4.1	-	Yes
	Klebsiella quasipneumoniae	INF291	R9.4.1	-	Yes
	Klebsiella variicola	INF022	R9.4	-	Yes
		KSB1-8J	R9.4.1	-	Yes
	Salmonella enterica	2010-06152	R9.4.1	-	Yes
	Acinetobacter baumannii	AYP-A2	R9.4	-	Yes
	Acinetobacter nosocomialis	MINF-5C	R9.4.1	-	Yes
	Acinetobacter ursingii	MINF-9C	R9.4.1	-	Yes
	Burkholderia cenocepacia	MINF-4A	R9.4.1	-	Yes
	Comamonas kerstersii	MSB1-7G	R9.4.1	-	Yes
	Haemophilus parainfluenzae	M1C146-1	R9.4.1	-	Yes
	Moraxella lincolnii	51409	R9.4	-	Yes
	Morganella morganii	MSB1-1E	R9.4.1	-	Yes
	Pseudomonas aeruginosa	MINF-7A	R9.4.1	-	Yes
	Stenotrophomonas pavanii	MSB1-4D	R9.4.1	-	Yes
[5]	*Mycobacterium tuberculosis*	-	R9.4	-	No
	Phage lambda	-	R9.4	-	No
	Escherichia coli	-	R9.4	-	No
[31]	*Escherichia coli*	-	R9	-	Yes
[17]	*Homo sapiens* - NA18943	NA18943	R9.4	-	No

Reference	Species	Dataset name	Pore version	Ligation kit	Available
[28]	*Homo sapiens* - NA12878	FAB23716	R9	Rapid	Yes
		FAB39088	R9.4	Ligation	Yes
		FAB39075	R9.4	Ligation	Yes
		FAB39043	R9.4	Ligation	Yes
		FAB42706	R9.4	Ligation	Yes
		FAB41174	R9.4	Ligation	Yes
		FAB42260	R9.4	Ligation	Yes
		FAB42804	R9.4	Ligation	Yes
		FAB42316	R9.4	Ligation	Yes
		FAB42205	R9.4	Ligation	Yes
		FAB42561	R9.4	Ligation	Yes
		FAB42473	R9.4	Ligation	Yes
		FAB42395	R9.4	Ligation	Yes
		FAB42476	R9.4	Ligation	Yes
		FAB42451	R9.4	Ligation	Yes
		FAB42704	R9.4	Ligation	Yes
		FAB42828	R9.4	Ligation	Yes
		FAB42810	R9.4	Ligation	Yes
		FAB42798	R9.4	Ligation	Yes
		FAB45280	R9.4	Ligation	Yes
		FAB46664	R9.4	Ligation	Yes
		FAB46683	R9.4	Ligation	Yes
		FAB45332	R9.4	Ligation	Yes
		FAB43577	R9.4	Ligation	Yes
		FAB44989	R9.4	Ligation	Yes
		FAF01169	R9.4	Ligation	Yes
		FAF01441	R9.4	Ligation	Yes
		FAB45277	R9.4	Ligation	Yes
		FAB45321	R9.4	Ligation	Yes
		FAF01127	R9.4	Ligation	Yes
		FAF01132	R9.4	Ligation	Yes
		FAB49712	R9.4	Ligation	Yes
		FAF01253	R9.4	Ligation	Yes
		FAB45321	R9.4	Ligation	Yes
		FAB49914	R9.4	Ligation	Yes
		FAB45271	R9.4	Ligation	Yes
		FAB49164	R9.4	Ligation	Yes
		FAB49908	R9.4	Rapid	Yes
		FAF04090	R9.4	Rapid	Yes
		FAF15665	R9.4	Ultra	Yes
		FAF13748	R9.4	Ultra	Yes
		FAF10039	R9.4	Ultra	Yes
		FAF09968	R9.4	Ultra	Yes
		FAF09277	R9.4	Ultra	Yes
		FAF14035	R9.4	Ultra	Yes
		FAF15694	R9.4	Ultra	Yes
		FAF09713	R9.4	Ultra	Yes
		FAF18554	R9.4	Rapid	Yes
		FAF15630	R9.4	Ultra	Yes
		FAF09640	R9.4	Ultra	Yes
		FAF09701	R9.4	Ultra	Yes
		FAF15586	R9.4	Ultra	Yes
		FAF05869	R9.4	Ligation	Yes
This publication	*Lambda Phage*	VER5940	R9.4.1	Ligation	Yes

Table S3: Datasets used for basecalling benchmarking. List of publicly released Nanopore sequencing datasets that have been used for benchmarking.

Species	Set	Difficulty
Klebsiella variicola	Train and test	0
Serratia marcescens	Train and test	0
Klebsiella quasipneumoniae	Train and test	0
Comamonas kerstersii	Train and test	0
Citrobacter koseri	Train and test	0
Klebsiella pneumoniae	Train and test	0
Enterobacter kobei	Train and test	0
Salmonella enterica	Train and test	0
Klebsiella aerogenes	Train and test	0
Morganella morganii	Train and test	0
Citrobacter freundii	Test	1
Shigella sonnei	Test	1
Escherichia coli	Test	1
Escherichia marmotae	Test	1
Burkholderia cenocepacia	Test	2
Stenotrophomonas pavanii	Test	2
Pseudomonas aeruginosa	Test	2
Moraxella lincolnii	Test	2
Acinetobacter ursingii	Test	2
Acinetobacter pittii	Test	2
Acinetobacter nosocomialis	Test	2
Acinetobacter baumannii	Test	2
Haemophilus haemolyticus	Test	2
Haemophilus parainfluenzae	Test	2
Staphylococcus aureus	Test	3
Homo sapiens	Test	4
Lambda phage	Test	4

Table S4: Species split in the cross-species task. Each species belongs to a bin depending on the k-mer genomic distance to the train-test species. Lower numbers indicate closer distance and higher numbers indicate further genomic distance.

	Basecalled reads (%)				Alignment events (%)				Hompolymer errors (%)				PhredQ scoring	
**Model**	Pass	Failed mapping	Short alignment	No prediction	Match	Mismatch	Insertion	Deletion	Adenine	Cytosine	Guanine	Thymine	Overlap	AUC
Bonito	93.4	**4.1**	2.3	0.2	90.0	**2.5**	2.6	4.3	12.5	14.9	19.7	15.8	32.4	0.910
Guppy original	**95.2**	4.4	**0.4**	**0**	**91.3**	**2.5**	2.0	**3.9**	**9.7**	**14.7**	**18.9**	**12.5**	27.9	**0.937**
Causalcall	66.3	25.7	7.8	0.2	77.6	6.0	**1.7**	14.4	43.4	60.0	50.7	42.0	**0.7**	0.802
Causalcall original	63.2	32.2	4.6	**0**	80.5	7.1	3.3	8.0	25.0	26.1	28.8	25.2	48.1	0.837
Halcyon	79.7	4.6	15.2	0.0004	81.1	3.3	3.2	8.7	20.8	20.0	22.0	25.9	11.7	0.844
Halcyon original	46.1	34.1	19.8	**0**	75.6	6.1	6.1	8.4	23.1	25.0	30.0	22.1	N/A	N/A

Table S5: Recreated models vs original models benchmark summary. Comparison of existing basecallers and our recreations based on the human task benchmark. Results in bold denote best performance.

Package name	Version
cupy-cuda102	9.4.0
fast-ctc-decode	0.3.0
h5py	3.7.0
koi-cuda102	0.0.5
mappy	2.21
matplotlib	3.4.2
numpy	1.21.5
onnxruntime	1.12.1
ont-fast5-api	4.0.2
ont-tombo	1.5.1
pandas	1.3.1
parasail	1.2.4
psutil	5.8.0
pysam	0.16.0.1
scikit-learn	0.24.2
scipy	1.7.1
seaborn	0.11.1
seqdist	0.0.3
torch	1.9.0
torchaudio	0.9.0
torchvision	0.10.0
tqdm	4.62.0

Table S6: Packages and versions used for model training and evaluation.
