# Supplementary material for: Comprehensive benchmark and architectural analysis of deep learning models for nanopore sequencing basecalling
Source: Genome Biol. 2023 Apr 11;24:71. doi: 10.1186/s13059-023-02903-2 (PMC10088207; doi:10.1186/s13059-023-02903-2)
Supplement: Supplementary file 2 — Additional file 2. The review history. [file 13059_2023_2903_MOESM2_ESM.docx]

**1^st^ round**

**Reviewer 1**

This paper does not directly benchmark existing basecallers. Rather, it takes the neural network architectures used in basecallers, reimplements them, retrains them using consistent data, and then benchmarks these models. This allows for consistent testing of the different architectures and training sets, and I believe it is a strength of the study. However, this distinction (e.g. testing Bonito itself vs testing the neural network architecture used in Bonito) is not always clear. For example: 'Bonito performed best in three out of the four metrics' on line 118 and 'we benchmarked the latest existing basecallers' on line 229. I therefore worry readers might misinterpret this study as a benchmark of the actual basecallers. To avoid confusion, I recommend being more explicit in the abstract (e.g. using the words 'reimplement' and 'retrain') and replacing basecaller names with a clearer description (e.g. using 'the Bonito-based model' instead of 'Bonito') throughout the paper.

The GitHub repository for the basecalling component of this study (github.com/marcpaga/basecalling_architectures) is almost completely lacking in documentation. Please add detailed instructions for how to train/basecall using this code for readers that would like to replicate the work or extend it using new models or data. This is especially important considering that the paper's abstract explicitly says that 'the community can further expand on this work'.

The other GitHub repository (github.com/marcpaga/nanopore_benchmark) does contain documentation, but it could use more. In particular, a thorough introduction section (which links to the paper) would be useful for explaining the purpose of the repository. The authors might also consider combining the two GitHub repositories into one for simplicity.

This manuscript focuses only on read-level accuracy and does not address consensus-level accuracy. E.g. a model might produce low accuracy reads, but if the errors were sufficiently random, the reads could still produce a consensus sequence with high accuracy. I'm not requesting that a consensus-level analysis be added to the study, but the scope should be clearly stated. I.e. the manuscript should make it clear that it is only addressing read-level accuracy, and a brief discussion of consensus-level accuracy is warranted in the conclusions.

This paper defined homopolymers as a sequence of the same base of length 5 or more. While this may be appropriate for the R9.4 reads used in this study, newer R10.4.1 pores from ONT can handle longer homopolymers. This threshold (5 bases) seems to be hard-coded in the evaluation.py script - can it be made adjustable? This would allow researchers to assess performance on homopolymers of length 6, 7, 8, etc.

**Reviewer 2**

In this manuscript, the authors systematically benchmarked recently proposed deep learning architectures for Nanopore basecalling.
They thoroughly analyzed the models' major components and effects on different evaluation metrics.
For fairness comparison, they curated benchmark datasets from previous works using R9.4/R9.4.1 pore chemistry for several species
and evaluated them in different task settings.
Their experiments demonstrate the effectiveness of complex convolutions, deeper RNN layers, and a CRF decoder.
In general, we appreciate such a third-party evaluation for the community, which can help to fill the gap of comparing updating nanopore basecallers and prompts an in-depth understanding of designing necessary model modules for basecalling.

For the manuscript, we have the following comments:

[Major comments]:

(1). For deep learning models, especially those running on GPUs, the randomness can affect the reproducibility and final models' performance.
Therefore, more detailed information on the computing environment (e.g., the CUDA version) and random seed settings are expected to be provided as much as possible. The authors have reported versions of used software and packages, while other detailed information is only partially found in the current manuscript and supplement.


(2). Homo sapiens and Lambda phage performance show a noticeable performance drop when compared with the bacteria datasets.
To explain this observation, the authors investigated the difficulty of basecalling on specific species (k-mer genomic distance between train-test dataset) and training data scale.
This is an interesting observation, but a more detailed analysis is expected to be provided (e.g., the read-length distribution and current-level analysis).
Other factors, such as different sequencing settings, should also be excluded, as the datasets are collected from multi-center sequencing.


(3). Although R9.4/R9.4.1 pore chemical nanopore data is the primary focus of this work, the new R10.3/R10.4 data is on the way.
How the findings of this work can be generalized in the new pore chemical data would be attractive to potential readers.


[Minor comments]:

(1). Typo in Paged 1 Line 24: "bus" -> "but".

(2). Section 2.2, Line 147: "Suggesting the decreased error might be due to the ...". It is confusing to understand the "decreased error", as a higher error rate is discussed in the context. Might the authors intend to express the "decreased performance"?

**Authors’ response**

Reviewer #1

1) This paper does not directly benchmark existing basecallers. Rather, it takes the neural network architectures used in basecallers, reimplements them, retrains them using consistent data, and then benchmarks these models. This allows for consistent testing of the different architectures and training sets, and I believe it is a strength of the study. However, this distinction (e.g. testing Bonito itself vs testing the neural network architecture used in Bonito) is not always clear. For example: 'Bonito performed best in three out of the four metrics' on line 118 and 'we benchmarked the latest existing basecallers' on line 229. I therefore worry readers might misinterpret this study as a benchmark of the actual basecallers. To avoid confusion, I recommend being more explicit in the abstract (e.g. using the words 'reimplement' and 'retrain') and replacing basecaller names with a clearer description (e.g. using 'the Bonito-based model' instead of 'Bonito') throughout the paper.

We agree that it is important that this distinction is clearly made throughout the paper. For this reason we have rewritten several parts of the paper so that it is clearer (including the two mentioned by the reviewer). We have also added an explicit sentence at the start of the benchmark results section stating that when mentioning a model by name we are referring to the architecture of the model. We have kept the model's names as they are, since we think adding the word implementation/architecture after every model name would make the text too verbose.

(2) The GitHub repository for the basecalling component of this study (github.com/marcpaga/basecalling_architectures) is almost completely lacking in documentation. Please add detailed instructions for how to train/basecall using this code for readers that would like to replicate the work or extend it using new models or data. This is especially important considering that the paper's abstract explicitly says that 'the community can further expand on this work'.

We apologize for the lack of documentation. We have now added extensive documentation throughout, including on how to train the models presented in this work, some demo data and the package requirements to reproduce the necessary environment.

(3) The other GitHub repository (github.com/marcpaga/nanopore_benchmark) does contain documentation, but it could use more. In particular, a thorough introduction section (which links to the paper) would be useful for explaining the purpose of the repository. The authors might also consider combining the two GitHub repositories into one for simplicity.

We have included an introduction to who might benefit from using this repository as well as links to the paper (bioRxiv for now) and also the architectures GitHub. We have also uploaded the Lambda phage data and updated the repository regarding that.

(4) This manuscript focuses only on read-level accuracy and does not address consensus-level accuracy. E.g. a model might produce low accuracy reads, but if the errors were sufficiently random, the reads could still produce a consensus sequence with high accuracy. I'm not requesting that a consensus-level analysis be added to the study, but the scope should be clearly stated. I.e. the manuscript should make it clear that it is only addressing read-level accuracy, and a brief discussion of consensus-level accuracy is warranted in the conclusions.

We agree that it should be made clear at which level this benchmark is addressed. We have added a clarifying sentence on the initial section of the results, as well as a brief comment on the discussion section regarding the randomness of the errors and its consequences on consensus-level basecalling.

(5) This paper defined homopolymers as a sequence of the same base of length 5 or more. While this may be appropriate for the R9.4 reads used in this study, newer R10.4.1 pores from ONT can handle longer homopolymers. This threshold (5 bases) seems to be hard-coded in the evaluation.py script - can it be made adjustable? This would allow researchers to assess performance on homopolymers of length 6, 7, 8, etc.

Thanks for the suggestion, indeed the homopolymer length was hardcoded as 5. We have updated the code on the benchmark GitHub so that the minimum length of a homopolymer is a user defined parameter that can be changed as a command line argument. We use a minimum length of 5 as the default parameter.

Reviewer #2:

In this manuscript, the authors systematically benchmarked recently proposed deep learning architectures for Nanopore basecalling. They thoroughly analyzed the models' major components and effects on different evaluation metrics. For fairness comparison, they curated benchmark datasets from previous works using R9.4/R9.4.1 pore chemistry for several species and evaluated them in different task settings. Their experiments demonstrate the effectiveness of complex convolutions, deeper RNN layers, and a CRF decoder. In general, we appreciate such a third-party evaluation for the community, which can help to fill the gap of comparing updating nanopore basecallers and prompts an in-depth understanding of designing necessary model modules for basecalling.

(1). For deep learning models, especially those running on GPUs, the randomness can affect the reproducibility and final models' performance. Therefore, more detailed information on the computing environment (e.g., the CUDA version) and random seed settings are expected to be provided as much as possible. The authors have reported versions of used software and packages, while other detailed information is only partially found in the current manuscript and supplement.

We thank the reviewer for bringing this to our attention. We have added details about the initialization of the random parameters on the methods section as well as a supplemental table with details of all the software packages as well as their versions used for the training of the models. We have also added details on the Python and CUDA version in the methods text as well as hardware requirements. We have also added a conda environment file with all the dependencies in the GitHub pages which allows complete reproduction of our results.

(2). Homo sapiens and Lambda phage performance show a noticeable performance drop when compared with the bacteria datasets. To explain this observation, the authors investigated the difficulty of basecalling on specific species (k-mer genomic distance between train-test dataset) and training data scale. This is an interesting observation, but a more detailed analysis is expected to be provided (e.g., the read- length distribution and current-level analysis). Other factors, such as different sequencing settings, should also be excluded, as the datasets are collected from multi- center sequencing.

We have performed additional analysis on the differences between the datasets by pooling the results of the 10 best models (as we do not see much difference in performance between them) and compared the characteristics of the human, bacterial and viral datasets. We have summarized the results in the main text as well as in a supplementary figure. In short, our main results are:

-There are differences in the read length distributions of the datasets; however, we do not observe any correlation between match rate and read length.

-Based on error profile analysis, we observe an increased error rate on human data at the 3-mers that contain a CG motif. We hypothesize that 5mC methylation is the likely cause of a higher error rate on those motifs, as this is also described in [https://journals.plos.org/plosone/article?id=10.1371/journal.pone.0257521].

-The human dataset also contains the largest amount of homopolymers, which would increase the difficulty to properly basecall them.

-We calculated difficulty scores by taking the weighted mean of the error rate of each 3mer by their abundance on the test set. We found that the bacterial dataset has a difficulty score of 0.8, while the virus and human have a 0.12 and 0.13 difficulty scores respectively. This means that on average, the latter contain more difficult 3-mers than the bacterial.

-We could not find any specific sequencing settings are mentioned in the publications of the datasets that we have collected; we therefore assume that the settings were the default ones.

(3). Although R9.4/R9.4.1 pore chemical nanopore data is the primary focus of this work, the new R10.3/R10.4 data is on the way. How the findings of this work can be generalized in the new pore chemical data would be attractive to potential readers.

We agree that R10.4 is slowly being adopted by the community as the new standard pore. This new pore has a different chemistry and motor protein, which will influence the raw signal. Until recently, buying R10.4 flow cells was reserved to a few early adopter labs, but nowadays these flow cells are available for everyone. Unfortunately, due to its recent introduction, the amount of publicly available R10.4 datasets that can be used for benchmarking is much less than for R9.4. Furthermore, R10.4 flow cells can be run at different temperatures to increase sequencing speed at the cost of accuracy, to achieve a proper comparison one would require of the same dataset sequenced at different temperatures. Notably, our benchmarking platform is agnostic of the pore type, so as soon as more data are available similar analyses can readily be made (by us and/or the community), not only to compare R9.4 to R10.4, but also for different R10.4 sequencing speeds. This is exactly the intention of our work: enabling and setting the standard for fair and unbiased comparison also for future new pore versions and DNN basecalling architectures.

(4). Typo in Paged 1 Line 24: "bus" -> "but".

Thanks for spotting the typo, it has been corrected.

(5). Section 2.2, Line 147: "Suggesting the decreased error might be due to the ...". It is confusing to understand the "decreased error", as a higher error rate is discussed in the context. Might the authors intend to express the "decreased performance"?

Thanks for spotting the mistake, it has been corrected.
